# Positive emotion dispositions and emotion regulation in the Italian population

**DOI:** 10.1371/journal.pone.0245545

**Published:** 2021-03-02

**Authors:** Alice Chirico, Michelle N. Shiota, Andrea Gaggioli

**Affiliations:** 1 Catholic University of the Sacred Heart of Milan, Milan, Italy; 2 Arizona State University, Tempe, Arizona, United States of America; 3 Applied Technology for Neuro-Psychology Lab, I.R.C.C.S. Istituto Auxologico Italiano, Milan, Italy; Aalborg University, DENMARK

## Abstract

The goal of this large-scale study was to test the relationship between positive emotion dispositions (i.e., Joy, Contentment, Pride, Love, Compassion, Amusement, and Awe) and two strategies of emotion regulation (i.e., reappraisal and suppression) in the Italian population. 532 Italian-speaking adults completed the Dispositional Positive Emotion Scales (DPES), the Positive and Negative Affective Schedule (PANAS), the Italian Emotion Regulation Questionnaire (ERQ), and the Big-Five Inventory (BFI). DPES scales showed high reliability. Exploratory Factor Analysis showed that a 6-factor model fits the Italian sample better. Joy and Contentment loaded on the same factor. Items assessing the other five emotions loaded on separate factors. The patterns of relationships between positive emotion dispositions, positive and negative affects traits (PANAS), and personality traits (BFI) indicated concurrent validity of the DPES. Twelve separated multiple regression models with BFI and ERQ factors as predictors and DPES factors as response variables showed that Extraversion significantly positively predicted of all DPES emotions. Agreeableness predicted Happiness, Love, Compassion, and Awe positively. Conscientiousness predicted Amusement and Love negatively and Compassion, Pride, and Happiness positively. Neuroticism predicted all emotions negatively except for Compassion. Positive emotions were significantly and positively predicted by reappraisal, and negatively predicted by suppression.

## Introduction

Evolution has provided us with a unique and valuable tool to respond adaptively to environmental changes through emotions [1, 2]. An increasing number of researchers conceive emotions as discrete, universal, evolutionary, functional, and organized system composed of behavioral, psychophysiological, experiential, and cognitive responses to perceived challenges and opportunities [3, 4]. Particularly, emotions organize the mind’s responses to specific adaptive situational demands [5]. Consequently, recurrent features of the situation have progressively acquired a place in the architecture of the emotional process [6]. For instance, sadness is usually triggered in response to a significant loss [7]; thus, it is also characterized by physiological withdrawal reactions to recover from the loss.

The recurrent need to manage a specific situation triggering the most fitness-promoting responses—relevant for adaptation—constantly shapes and refines emotion-related features. Therefore, these regulatory processes vary according to a given emotion [e.g., 8]. In the first decade of the twentieth century, several researchers have started a new line of research focusing on the link between specific *discrete emotions* and their management, that is, peculiar *emotion regulation* (ER) strategies [e.g., 9, 10]. They showed differential emotion regulation paths, especially for each negative emotion, such as sadness [8] or shame [10]. However, although the interest in regulatory processes and discrete negative emotions-link has increased recently, regulation strategies occurring within the unfolding of positive emotions are still an open issue, maybe, since benefits associated with positive emotions are slower to come [11], while detrimental effects of negative emotions are immediately evident and urgent [9, 12]. Specifically, positive emotions have been mainly conceived as desirable phenomena that people attempt to maintain or enhance [19].

Only recently, an increasing number of researchers [e.g., 5, 6, 13] have devoted their attention to the relationship between other general positive (vs. negative) affect and specific emotion regulation strategies, such as *cognitive reappraisal* and *expressive suppression*. These two strategies can be deployed at the two poles of emotion regulation process, thus, virtually covering the full process of an emotional response. *Cognitive reappraisal* comes early and affects the onset of an emotional response, and it involves re-thinking a situation to change its meaning and emotional effect [14]. On the other hand, *Suppression* is a response-focused strategy that moderates outward signs of an emotional response, already deployed and appraised, by decreasing concurrent emotion-expressive behavior [15]. Cognitive reappraisal displayed a healthier pattern of emotional, cognitive, and social effects compared to expressive suppression [16]. Trait cognitive reappraisal predicted reactivity to affective stimuli by increasing the positive effect and decreasing the negative one [14, 17]. On the other hand, trait suppression appraisal led to more negative affective states [18]. Crucially, Barrett et al. [9] found that individuals who were better able to distinguish discrete emotional states were also more competent in regulating negative and positive emotions compared to those who were less able to differentiate specific discrete emotions. In conclusion, fairly little is known about how these two strategies interact with specific positive emotions, which are crucial for our flourishing [19], and nothing has been investigated regarding cross-cultural variations of this link.

Take the case of compassion. It should be conceived as a positive emotion serving specific interpersonal functions [20–22]. Compassion can be defined as a “feeling that arises in witnessing another’s suffering and that motivates a subsequent desire to help” [23]. It entails a commitment to and thus facilitation of a relationship [24]. Therefore, this emotion should be downregulated to save resources and direct them only to specific people [25, 26]. This would be far more crucial if dealing with a stable tendency to experience compassion related to stable or recurrent circumstances, as suggested by Goetz, Keltner [23]. With this regard, an emerging trend in emotion theories suggests that positive emotions should be considered not only as discrete states evolutionarily shaped to respond to specific demands [20, 24, 27–29] but also as stable traits or dispositions to specific eliciting situations.

Daily life is imbued with systematic attempts to modify even specific positive emotions that do not fit the social norms of a situation. For instance, we try to avoid an amused laugh every time our colleague we dislike shows up. We try not to exhibit an excessive proudful smile every time we beat all other competitors at work. We attempt to empathize when our tenderhearted friend talks about her new boyfriend at dinner. These eliciting situations can repeatedly occur, thus bringing forth stable emotional tendencies.

However, while the relationship between discrete dispositional positive emotions and other durable tendencies, such as personality traits [e.g., 30] or specific strengths and virtues [31], has been deeply investigated, the way people usually regulate them has remained unexplored. Specifically, research has showed that in the domain of stable affect, not all emotion regulation strategies can be considered as equal [32]. Despite this evidence, nothing is known about the unique pathway linking specific emotion regulation strategies to specific discrete positive emotions, whose peculiar functional value it is crucial to support people’ wellbeing and health [33].

### Measuring the disposition to experience positive emotions

In line with this evolutionary-functionalist approach on dispositional positive emotions, Shiota et al. [20], developed a self-reported instrument to assess individuals’ proneness to experience seven discrete positive emotional states, namely, Joy, Contentment, Pride, Love, Compassion, Amusement, and Awe. *Joy* is usually associated with a sense of strength and self-confidence [34] in safe and secure situations that promote the exploitation of new resources [35] and social connections [36]. *Contentment* is a low-arousal emotion related to the evaluation of actual material resources as exceeding individuals’ needs. This emotion invites people to savor their time while becoming aware of the available resources that can be integrated into schemas on self and others [37]. Therefore, *Contentment* can change both worldview and self-perception. *Pride* is a self-conscious emotion [38] arising from success during a socially relevant activity [20]. It increases our status, provides information about the level of social acceptance, and expands the possibility to exploit more group resources [20, 39]. Shiota et al. [20] defined *Love* based on Bowlby’s attachment behavioral system [40, 41]. People perceive a positive emotion of closeness towards trustworthy and dependable persons who take care of them. *Compassion* consists of feelings of concern and nurturance towards other people, even non-kin [42], especially if they are children, helpless, and vulnerable [e.g., 43–45]. Love and compassion facilitate social connectedness [20]. *Amusement* arises when there is a shift between one’s expectancies and one’s experience, and it usually occurs during humoristic situations [46]. Finally, *Awe* is a complex emotion arising when faced with vast and cognitively challenging stimuli [47]. Awe is a prosocial [48], epistemological [24, 47], and self-transcendent emotion [49].

Shiota et al. [20] showed that each positive emotion disposition of this scale correlates significantly with at least one of the BFI dimensions (i.e., Extraversion, Agreeableness, Conscientiousness, Neuroticism, and Openness to Experience), in line with the broadening nature of positive emotions [50]. The only exception was Compassion, which showed a small and not significant correlation with Neuroticism. Albeit preliminary, these findings are in line with the assumption that personality traits could predict specific emotional tendencies. Moreover, studies on dispositional discrete positive emotions have been conducted mainly in English-speaking countries. Thus, although DPES was developed to capture potentially universal, functionally distinct, and discrete emotion constructs, as well as their relationship with other universal phenomena, the definitions of each emotion reflect English-language constructs. Therefore, it is relevant to test the DPES factor structure also in Italian, among other languages.

In backing up this claim, a six-factor solution (instead of the original seven-factors structure) emerged from the exploratory analysis in the German validation of the scale, which was carried out at the item level [31] and in a more recent validation study carried out by Dixson, Anderson, & Keltner [51]. Specifically, Contentment and Joy loaded on the same factor, while other emotions loaded on the residual five factors. This study showed that emotions could be split into two subtypes. Pride, Joy, and Contentment appeared to reflect *self-oriented* emotions while Amusement, Love, Compassion, and Awe emerged as *other-focused* emotions, depending upon their main elicitors. In this regard, the evolutionary functionalist account suggests that emotions emerge quickly from specific eliciting situations and serve to manage them–usually–in the most appropriate way [14, 15, 52]. Since different emotions arise from different elicitors and trigger different action tendencies, it has been suggested that specific strategies could regulate each emotional state [53–55]. These strategies, which help people redirect the instinctive emergence of emotions, are considered the components of the “emotion regulation” process [14]. However, to our best knowledge, no study has investigated the link between discrete positive emotions and specific emotion regulation strategies.

Therefore, it becomes far more relevant to investigate whether the frequent use of a given emotion regulation strategy is associated with specific positive emotions and whether it can, eventually, predict them. In this study, we sought to investigate the link between specific discrete positive emotions and two main categories of emotion regulation strategies of emotion-regulation, that is, cognitive reappraisal and expressive suppression. Thus, we aimed to determine whether there can be differential paths linking the two emotion regulation categories and each positive emotion. We also aimed to test whether specific BFI factors could predict each discrete emotion disposition. In the following paragraph, we detail the direction of the causal link between emotion regulation strategies and dispositions to experience positive emotions.

### The current study

To contribute to the current literature on emotion regulation and discrete positive emotions, we carried out an exploratory study aimed at investigating the relationship between the seven discrete positive emotions of DPES and two major emotion regulation strategies, namely, Reappraisal and Suppression [15, 18]. With this regard, while the relationship between discrete dispositional positive emotions and other durable tendencies, such as personality traits [e.g., 30] or specific strengths and virtues [31], has been deeply investigated, the way people usually regulate them has remained unexplored. Despite it has been demonstrated the unique predictive value of specific emotion regulation strategies on stable affect [32], nothing is known about the unique pathways linking specific emotion regulation strategies to specific discrete positive emotions considered as stable traits, whose peculiar functional value it is crucial to support peoples’ wellbeing and health [33].

In this study, the DPES by Shiota et al. [20] and the Italian version of the Emotion Regulation Questionnaire (ERQ) [18] were administered to a sample of native Italian participants. ERQ assesses how frequently individuals use cognitive reappraisal or suppression strategies. Given the high degree of differentiation among discrete positive emotions [20], we hypothesized the existence of different correlation patterns between each emotion and each emotion regulation strategy, which can be explained in light of a functional model of emotions. Specifically, starting from evidence on the impact of peculiar ER strategies on stable affect and emotions [19, 32, 56–58], we assumed that the frequent use of a given emotion regulation strategy (cognitive reappraisal or suppression) could predict also discrete stable positive emotions.

Moreover, since the definitions of each emotion reflected English-language constructs, as mentioned earlier, we also tested the factor structure of the translated Italian version of the DPES, as well as its concurrent and divergent validity. We also administered the Italian version of Positive and Negative Affect Schedule [59] as a measure of concurrent validity concerning the DPES (see [51]. PANAS is a well-validated measure of positive vs. negative affect in Italian. Finally, we also assessed the Big Five personality traits using the Big Five Inventory (BFI) [60] Italian version [61]. Therefore, the concurrent validity of the Italian DPES was studied by examining both the extent to which the different scales were associated with the two global dimensions of affect, as assessed by PANAS, and with Big Five factors, as assessed by the BFI Italian version. In line with previous validation studies [20, 31, 62], we expected positive correlations of all DPES factors with PANAS scales, as well as with Extraversion and Openness to Experience personality traits, and negative correlations with Neuroticism trait. Moreover, given preliminary but promising evidence on the link between BFI and DPES factors [30], we tested also the contribution of BFI factors to each discrete emotion.

## Method

### Participants

The Italian DPES version was administered to 532 participants from Italy (mean age = 25.2; SD = 5.698, range = 18–49) of whom 439 were women (mean age = 25.05; SD = 5.448) and 93 were men (mean age = 26.42; SD = 6.671). Sample size was established on the base of previous works that validated or used scale in US or European populations [20, 31, 51, 62]. Their religious orientation was as follows: 63.62% was Catholic, 0.38% was Buddhist, 0.56% were Muslim or Protestant, 34.77% indicated practicing a non-conventional religion or being Atheists. Mean years of education of the sample was 16.20 (S.D. = 2.812), ranging from a minimum of 5 to a maximum of 26 years. Mean years of education was 15.58 (S.D. = .310) for men, ranging from 8 to 22 years, and 16.32 (S.D. = .133) for women, ranging from 5 to 26 years. The participants were recruited through social networks and flyers. They completed the online version of the DPES voluntarily and received no credit for their participation in the study. The same participants also completed the Italian Emotion Regulation Questionnaire [18], Positive and Negative Affect Schedule Italian version [59], and the BFI Italian version [60] on the same occasion. All the instruments are reported and described in the next section “Materials”. The study was approved by the Ethical Committee of the Università Cattolica del Sacro Cuore of Milan prior to data collection and it was conducted in accordance with the Helsinki Declaration.

### Materials

In this study, the Italian version of the DPES was translated from the original English questionnaire [20], and it uses the same item numbering. The DPES is a self-administered questionnaire, composed of 38 items that measure the proneness to live seven discrete positive emotions. It was structured by seven different scales, each representing a specific positive emotion: Joy (6 items), Contentment (5 items), Pride (5 items), Love (6 items), Compassion (5 items), Amusement (5 items), and Awe (6 items). The 38 items were presented to responders in a seven-step Likert scale ranging from Strongly Disagree (1) to Strongly Agree (7). First, two bilingual translators (one expert in the field of emotions and the other naïve) translated Dispositional Positive Emotions to Italian, as suggested by Borsa, Damásio and Baindeira [63]. Further, the accuracy of the translation was tested by a back-translation from Italian to English done by two different bilingual translators, both fluent in Italian and English. The back-translation was checked by one of the authors of the original version. Afterward, three expert judges compared the original and back to finalize the Italian version.

### Italian version of the ERQ

Italian ERQ [18] is a 10-item 7-points likert (1 = strongly disagree; 7 = strongly agree) scale consisting into two scales each measuring an emotion regulation strategy: suppression (4 items) and reappraisal (6 items). Participants were instructed to think about how they control (that is, regulate and manage) their emotions. Cronbach alpha for both original scales was moderate to high (reappraisal = .84; suppression = .72).

### Italian version of the PANAS

The Italian PANAS version [59] captures the two main clusters of the affective experience. Specifically, this questionnaire is composed of 20 adjectives measuring the two main dimensions of affective experience: the positive (10 adjectives) [Positive Affect scale (PA)] and the negative one (10 adjectives) [Negative Affect (NA)]. In this study, we adopted the trait version by instructing participants to report the extent to which they usually feel as described by each of the 20 adjectives. The Italian PANAS version showed an adequate internal-consistency reliability for PA (.83) and for NA (.87) using the trait time instruction.

### Italian version of the BFI

The Italian Big Five Inventory (BFI) [60] is a is a self-report questionnaire relying on the work of [64], which measures the five personality trait dimensions defined by the Five-Factor Theory of Personality: Conscientiousness (9 items), Agreeableness (9 items), Extraversion (8 items), Neuroticism (8 items), and Openness (10 items). It consists of 44 items on a 5-point Likert scale, from 1 “Disagree Strongly” to 5 “Strongly Agree”. The internal consistency range in this sample was α = 0.70−0.84.

### Procedure

Participants completed the survey in Qualtrics survey software and were provided with an online informed consent. No minors were included. Data have been anonymized. Participants were fully informed regarding the procedure and objectives of this study. They were recruited through flyers, social networks and by word of mouth. They were first informed that they would be completing some questionnaires about positive and negative emotions. Then, first, they filled the first part of the questionnaire created to gather socio-demographic information. Secondly, they completed the Italian translation of the DPES. Finally, they completed the Italian version of the PANAS and of the BFI.

## Results

This study was designed to test the relationship between two emotion regulation strategies and seven emotion dispositions. This was the first time that this scale was administered to an Italian sample. Therefore, we first assessed the structure of the DPES to determine the number of components needed to adequately describe the psychological constructs of Positive Emotions Dispositions in the Italian sample. We then assessed concurrent and divergent validity with PANAS and BFI subscales. Finally, we examined the relationship between the two emotion regulation strategies and the resulting emotion dispositions.

### Primary analyses

To examine the structure of the Italian DPES, an Explorative Factor Analysis (EFA) analysis was carried out on the original set of items. Data were analyzed by IBM SPSS Statistics software (Version 21, release 21.0.0.0 64-bit edition). No missing values were found. Skewness and kurtosis of DPES items indicated a normal distribution. The means of factors ranged from 5.704 (compassion) to 4.420 (love). To get a clear idea of the relationships between the seven emotional dispositions and their underlying latent constructs, we carried out an EFA. The aim was to uncover the underlying structure of this large set of variables (38 items) in an Italian sample [65].

### Structure of the DPES scale: Exploratory factor analysis

Before carrying out the Exploratory Factor Analysis, a parallel Monte Carlo simulation analysis [66] was run on the 38 items to determine the number of factors to retain in EFA. This analysis revealed a maximum of seven-factor structure. The seven-factor solution was confirmed by the eigenvalues, indicating that 7 factors had eigenvalues greater than 1.0, which accounted for 36.0% of the total variance. Inspection of the correlation matrix showed several coefficients of 0.30 and above.

Therefore, we chose to test the 7-factor solution in line with the original structure of the scale, as suggested by Parallel Analysis. Moreover, given the nature of the scale, which assesses positive emotions, we expected that our factors were correlated. Therefore, first, we carried out a Principal Axis Factoring (PAF) analysis with oblimin rotation (delta = 0). Pearson’s r correlations among factors were closer to .3. The 7-factor solution explained 60.301% variance. This solution was also supported by Kaiser-Meyer-Olkin Measure of Sampling Adequacy (.90) and Bartlett’s test of Sphericity [χ2 (703) = 9818.071; p < .01]. However, item 36 had a very low level of communality (.106) and items did not load clearly in the seven factors; therefore, we chose to trim item 36, and we ran the PAF again, with oblimin rotation forcing a 7-factor solution. Global explained variance increased (61.481%). However, most items still double-loaded on several factors, and the resulting factorial structure was not easily interpretable. Therefore, we chose to run PAF again, with oblimin rotation forcing a 6 factors solution, in line with the findings of Gusewell and Ruch [31] (see Table 1 for Factors correlation matrix using principal axis factoring with oblimin rotation 6-factor solution).

**Table 1:** Factors correlation matrix using principal axis factoring with oblimin rotation 6-factor solution.

Factor	1	2	3	4	5	6
1	1.000	.073	.064	.376	.382	.376
2	.073	1.000	.233	.234	.097	.198
3	.064	.233	1.000	.174	.058	.170
4	.376	.234	.174	1.000	.222	.231
5	.382	.097	.058	.222	1.000	.178
6	.376	.198	.170	.231	.178	1.000

This solution explained less variance (58.398%), but all items loaded on each factor more clearly, without double loading items or just one item per factor. Specifically, the final solution had all Joy (i.e., 1, 2, 3, 4, 5, 6) and Contentment (i.e., 7, 8, 9, 10, 11) items loading on the first factor, while the Pride, Love, Compassion, Humor, and Awe items each loaded on separate factors. Only items from the original Joy scale merged with those of the original Contentment scale, while Pride emerged as a separate factor. This is in line with the validation study of the German version of this scale by Güsewell and Ruch [31]. Consistent with Güsewell and Ruch [31], we selected the 6-factors solution as the best model to explain our variables (see Table 2. for the final factor solution).

**Table 2:** Final factor solution: Pattern matrix showing factor loadings from exploratory factor analysis PAF with oblimin rotation in the final version of the scale (37 items, n = 532).

Items	Happiness	Compassion	Amusement	Love	Pride	Awe
1. **I often feel bursts of joy** (Provo spesso esplosioni di gioia)	**0.644**	0.07	0.048	0.086	-0.087	0.135
2. **I am an intensely cheerful person** (Sono una persona profondamente allegra)	**0.785**	0.062	0.299	0.042	0	-0.111
3. **I am often completely overjoyed when something good happens**. (Sono spesso completamente pieno di gioia quando accade qualcosa di buono)	**0.599**	0.321	-0.014	0.029	-0.058	0.019
4. **On a typical day, many events make me happy** (In una giornata tipo. ci sono molte cose che mi rendono felice)	**0.559**	0.006	-0.05	0.16	0.055	0.21
5. **Good things happen to me all the time** (Mi accadono continuamente cose positive)	**0.449**	-0.12	-0.043	0.148	0.18	0.271
6. **My life is always improving** (La mia vita è in continuo miglioramento)	**0.389**	-0.067	-0.129	0.009	0.289	0.286
7. **I am generally a contented person** (Generalmente sono una persona contenta)	**0.667**	-0.048	0.105	0.083	0.12	0.02
8. **I am at peace with my life** (Sono in pace con me stesso)	**0.454**	-0.133	-0.095	0.012	0.409	0.023
9. **When I think about my life I experience a deep feeling of contentment** (Quando penso alla mia vita sento un profondo senso di appagamento).	**0.513**	-0.099	-0.072	0.027	0.428	0.106
10. **I feel satisfied more often than most people** (Mi sento soddisfatto più spesso della maggior parte delle persone)	**0.428**	-0.139	0.066	0.117	0.253	0.158
11. **My life is very fulfilling** (La mia vita è molto gratificante)	0.425	-0.062	-0.107	0.036	**0.475**	0.167
12. **I feel good about myself** (Mi sento soddisfatto di me stesso)	0.271	-0.04	-0.1	-0.001	**0.695**	0.037
13. **I am proud of myself and my accomplishments** (Sono orgoglioso di me stesso e dei miei traguardi)	0.168	0.019	-0.127	-0.006	**0.67**	-0.01
14. **Many people respect me** (Molte persone mi rispettano)	-0.038	0.089	0.1	0.191	**0.514**	-0.069
15. **I always stand up for what I believe** (Mi batto sempre per quello in cui credo)	0.022	0.351	0.057	-0.061	**0.375**	0.006
16. **People usually recognize my authority** (Le altre persone di solito riconoscono la mia autorità)	-0.136	0.071	0.16	0.065	**0.553**	0.019
17. **Other people are generally trustworthy** (Le persone sono per lo più degne di fiducia).	-0.014	-0.045	-0.127	**0.658**	0.052	0.164
18. **I develop strong feelings of closeness to people easily** (Io sviluppo facilmente una forte intimità con le persone)	-0.037	0.086	0.116	**0.597**	0.046	-0.058
19**. I find it easy to trust others** (Trovo semplice fidarmi degli altri).	-0.072	-0.11	0.035	**0.864**	-0.071	0.063
20**. I can depend on people when I need help** (Sono in grado di affidarmi agli altri quando ho bisogno d’aiuto).	0.016	0.072	-0.02	**0.585**	0.008	-0.082
21. **People are usually considerate of my needs and feelings** (Di solito le persone sono attente ai miei bisogni e ai miei sentimenti)	0.12	-0.049	0.003	**0.537**	0.09	-0.03
22. **I love many people** (Voglio bene a molte persone)	0.094	0.22	0.032	**0.478**	-0.028	0.023
23. **It’s important to take care of people who are vulnerable** (È importante prendersi cura delle persone che sono vulnerabili)	-0.092	**0.592**	0.027	0.158	0.008	0.05
24**. When I see someone hurt or in need, I feel a powerful urge to take care of them** (Quando vedo persone ferite o che hanno bisogno. sento un forte impulso a prendermi cura di loro)	0.01	**0.858**	0.007	-0.045	0.056	-0.015
25. **Taking care of others gives me a warm feeling inside** (Prendermi cura degli altri mi procura una sensazione piacevole).	0.061	**0.796**	-0.089	0.008	-0.01	0.067
26. **I often notice people who need help** (Spesso noto le persone che hanno bisogno di aiuto)	-0.031	**0.643**	0.002	-0.006	0.096	0.068
27. **I am a very compassionate person** (Sono una persona molto compassionevole)	0.083	**0.606**	0.022	0.038	-0.066	0.082
28**. I find humor in almost everything** (Trovo dell’umorismo in quasi ogni cosa).	-0.025	-0.067	**0.681**	0.018	0.052	0.155
29. **I really enjoy teasing people I care about** (Mi piace molto prendere bonariamente in giro le persone alle quali tengo.	-0.052	0.039	**0.636**	-0.094	0.081	-0.053
30. **I am very easily amused** (Sono una persona che si diverte con facilità)	0.301	0.037	**0.549**	0.119	-0.003	0.032
31. **The people around me make a lot of jokes** (Le persone attorno a me scherzano spesso)	0.121	0.091	**0.428**	0.104	-0.039	-0.016
32. **I make jokes about everything** (Io scherzo su ogni cosa)	0.026	-0.113	**0.774**	-0.01	-0.048	0.125
33. **I often feel awe.** (Provo spesso un senso di profonda meraviglia e soggezione)	0.139	0.148	0.05	0.034	-0.157	**0.582**
34. **I see beauty all around me** (Percepisco la bellezza tutt’attorno a me)	-0.004	0.084	0.049	0.114	-0.024	**0.75**
35. **I feel wonder almost every day** (Provo meraviglia quasi tutti i giorni)	0.103	-0.006	0.04	0.025	-0.046	**0.795**
37. **I have many opportunities to see the beauty of nature** (Ho molte opportunità di vedere la bellezza della natura)	0.004	0.024	-0.045	0.049	0.016	**0.567**
38. **I seek out experiences that challenge my understanding of the world** (Io ricerco esperienze che sfidano la mia visione del mondo)	-0.093	0.036	0.097	-0.093	0.049	**0.455**

*Note*. All original items from Shiota et al. [20] are in bold.

Items loading on **Factor 1**, which we called “Happiness” (ten items), represent a positive attitude towards life in general (e.g., “I am generally a contented person”). This factor encompasses all items from the original “Contentment” scale (except for item 11 that loaded on Factor 5) as well as items from the original “Joy” scale (e.g.., “Good things happen to me all the time”; “My life is always improving”). Items loading on **Factor 2**, called “Compassion” (five items), represent a desire to take care of, or a concern for, another’s well-being, especially for those in need of help and vulnerable. Items loading on Compassion factors are the same as those loading on the original scale. Items loading on **Factor 3**, “Amusement” (five items), refer to positive feelings in humorous situations perceived as inconsistent with one’s expectations [46]. Items loading on **Factor 4**, called “Love” (six items), are the same as the original factors called “Love.” This factor concerns trust towards other people who are conceived as straightforward and authentic. Items loading on **Factor 5**, called “Pride” (six items), the same items as those in the original scale along with the last item from the original “Contentment” scale (item: “My life is very fulfilling), capture a social recognition of self-identity (e.g., “Many people respect me”). Finally, items loading on **Factor 6** “Awe” (five items) all belong to the original scale. This Factor refers to a deep sense of wonder and astonishment when facing something grand to an extent to require an update of individuals’ current mental frames [47]. Only original item 36 was omitted due to the low response variance (i.e., “I often look for patterns in the objects around me”).

### Gender differences for DPES factors in the Italian sample

We also tested gender differences in each DPES factor, since Güsewell and Ruch [31] found significant differences between men and women in joy, contentment, compassion, and awe. A one-way ANOVA with gender as factor showed that women (mean = 5.75; S.D. = .791) scored significantly higher compared to men on compassion (mean = 5.46; S.D. = .982) [F(1) = 9.468; p = .002; partial eta square = .018], while men reported significantly higher levels of amusement (mean = 5.24; S.D. = .904) compared to women (mean = 4.928; S.D. = .996) [F(1) = 7.861; p = .005; partial eta square = .018].

### Scale reliabilities and concurrent validities

A further aim of this study was to assess the internal consistency and reliability of the DPES scales. We calculated the Cronbach’s alpha coefficients for all scales. Internal consistency of each scale was high: “Happiness” scale (.906), Pride scale (.798), and the Awe scale (.794), with .80 for love, .80 for compassion, and .75 for amusement. The Awe scale and the overall reliability increased compared to the original scale. This study also investigated the criterion validity of the scale, correlating it first with the two subscales of PANAS, which are self-report measures conceptually similar, assessing the same construct of experiencing positive emotions. Second, the correlations between DPES, BFI and ERQ scores were computed. The internal consistency and criterion validities were calculated using the data from the 532 participants. All correlations between DPES, BFI, and ERQ, as well as PANAS, were carried out considering the Italian structure of DPES (i.e., 6-factors structure).

#### PANAS

Reliability was calculated using the data from the 532 participants. The scale showed high reliability (Cronbach’s Alpha = .911). To calculate the scale criterion validity, we computed a Pearson correlation between the DPES and the PANAS positive and negative subscales scores (i.e., PA- Positive Affect; NA- Negative Affect). In this sample, the PA subscale of the PANAS had good reliability (Cronbach’s Alpha = .839), as did the NA subscale (Cronbach’s Alpha = .881).

Factors from the DPES correlated positively with the Positive Affect Scales and negatively, or not at all, with the Negative Affect Scale. Most correlations were significant (See Table 3).

**Table 3:** Pearson correlations between PANAS factors (Positive–PA- and Negative–NA–Affect) and DPES factors.

Variables	Happiness	Pride	Love	Compassion	Amusement	Awe
NA	-.425**	-.358**	-.173**	.016	.023	-.033
PA	.485**	.496**	.138**	.228**	.251**	.383**

*Note*.

* = p < .05.

** = p < .01.

### BFI

Reliability was calculated using the data from the 532 participants. The BFI scale showed a good level of reliability (Cronbach’s Alpha = .783). To calculate the scale criterion validity, we computed Pearson correlations between the DPES and the BFI subscales scores (i.e., Extraversion, Agreeableness, Conscientiousness, Neuroticism, Openness). Correlations are reported in Table 4.

**Table 4:** Pearson correlations between BFI factors (Extraversion, Agreeableness, Conscientiousness, Neuroticism, Openness) and DPES factors.

Variables	Happiness	Pride	Love	Compassion	Amusement	Awe
Extraversion	.461**	.463**	.435**	.237**	.259**	.247**
Agreeableness	.343**	.169**	.563**	.340**	.038	.238**
Conscientiousness	.249**	.436**	-.015	.091*	-.170**	.058
Neuroticism	-.482**	-.338**	-.189**	.153**	-.096*	-.136**
Openness	.160**	.205**	.099*	.231**	.149**	.452**

*Note*.

* = p < .05.

** = p < .01.

Finally, all emotions displayed significant positive correlations with Openness to experience personality factors. Given the correlations between discrete positive emotions of DPES and BFI factors, we chose to carry out six multiple linear regression models with Extraversion, Agreeableness, Conscientiousness, Neuroticism, and Openness as predictors and each discrete emotion as the response variable. The results are reported in Table 5.

**Table 5:** Summary of multiple regression analyses for BFI factors predicting each discrete emotion (n = 532).

	Happiness					Pride					Love					Compassion					Amusement					Awe				
			95% Confidence Interval					95% Confidence Interval					95% Confidence Interval					95% Confidence Interval					95% Confidence Interval					95% Confidence Interval		
Predictor	*B*	*SE B*	*Lower*	*Upper*	*p*	*B*	*SE B*	*Lower*	*Upper*	*p*	*B*	*SE B*	*Lower*	*Upper*	*p*	*B*	*SE B*	*Lower*	*Upper*	*p*	*B*	*SE B*	*Lower*	*Upper*	*p*	*B*	*SE B*	*Lower*	*Upper*	*p*
Extraversion	0.054	0.006	0.042	0.066	**< .001**	0.051	0.005	0.04	0.06	**< .001**	0.062	0.006	0.05	0.074	**< .001**	0.025	0.006	0.013	**0.036**	**< .001**	0.044	0.007	0.029	0.058	**< .001**	0.016	0.007	0.002	0.03	**0.022**
Agreeableness	0.033	0.006	0.020	0.045	**< .001**	-2.96e4	0.006	-0.011	0.01	0.958	0.095	0.007	0.08	0.108	**< .001**	0.05	0.006	0.038	0.062	**< .001**	-0.006	0.008	-0.021	0.009	0.452	0.025	0.007	0.011	0.04	**< .001**
Conscientiousness	0.016	0.006	0.006	0.027	**0.003**	0.046	0.005	0.037	0.011	**< .001**	-0.022	0.006	-0.033	-0.011	**< .001**	0.009	0.005	-0.002	0.019	0.105	-0.039	0.007	-0.051	-0.025	**< .001**	-0.005	0.006	-0.018	0.008	0.444
Neuroticism	-0.061	0.006	-0.073	-0.049	**< .001**	-0.03	0.005	-0.04	0.0111	**< .001**	-0.007	0.006	-0.019	0.005	0.232	0.039	0.006	0.028	0.051	**< .001**	-0.016	0.007	-0.03	-0.001	**0.036**	-0.015	0.007	-0.029	-8.35e−4	**0.038**
Openness	0.004	0.005	-0.007	0.015	0.513	0.01	0.005	4.69e-4	0.02	**0.04**	-0.011	0.006	-0.022	-7.51e−8	**0.050**	0.018	0.005	0.007	0.028	**0.001**	0.017	0.008	0.003	0.03	**0.013**	0.067	0.007	0.054	0.079	**< .001**
*R*^*2*^	.411					.386					.437					.229					.131					.248				
Adj *R*^*2*^	.405					.380					.431					.222					.123					.241				
*F*(5,526*)*	73.273					66.139					81.606					31.302					15.842					34.692				

*Note*. N = 532. Only three decimals were reported for each value in the table. Significant results are reported in bold.

Given the correlations between discrete positive emotions of DPES and ERQ factors (Table 6.), we chose to carry out six multiple linear regressions to test whether trait cognitive reappraisal and trait suppression significantly predicted participants’ ratings of each positive emotion disposition. See Table 7 for all multiple regression analyses with DPES factors as measures and each emotion regulation strategy (Cognitive Reappraisal and Suppression) as predictors.

**Table 6:** Pearson correlations between ERQ factors (Suppression and reappraisal) and DPES factors.

Variables	Happiness	Pride	Love	Compassion	Amusement	Awe
Suppression	-.194**	-.290**	-.306**	-.144**	.026	-.056
Reappraisal	.305**	.227**	.105*	.141**	.185**	.300**

*Note*.

* = p < .05.

** = p < .01.

**Table 7:** Summary of single regression analyses for each discrete emotion predicted by cognitive reappraisal and suppression as factors (n = 532).

	Happiness					Pride					Love					Compassion					Amusement					Awe				
			95% Confidence Interval					95% Confidence Interval					95% Confidence Interval					95% Confidence Interval					95% Confidence Interval					95% Confidence Interval		
Predictor	*B*	*ES B*	*Lower*	*Upper*	*P*	*B*	*SE B*	*Lower*	*Upper*	*p*	*B*	*SE B*	*Lower*	*Upper*	*p*	*B*	*SE B*	*Lower*	*Upper*	*p*	*B*	*SE B*	*Lower*	*Upper*	*p*	*B*	*SE B*	*Lower*	*Upper*	*p*
Cognitive Reappraisal	0.0452	0.006	0.033	0.057	**< .001**	0.0301	0.005	0.020	0.0401	**< .001**	0.0178	0.214	0.006	0.03	**< .001**	0.018	0.005	0.008	0.028	**< .001**	0.027	0.00615	0.0144	0.0195	**< .001**	0.045	0.007	0.033	0.057	**< .001**
Suppression	-0.039	0.008	-0.054	-0.024	**< .001**	-0.06	0.007	-0.063	-0.0367	** .001**	-0.062	0.00819	-0.078	-0.046	**0.004**	-0.024	0.007	-0.037	-0.0106	**< .001**	0.004	0.008	-0.012	0.039	0.661	-0.014	0.008	-0.029	0.003	0.097
*R*^*2*^	0.136					0.142					0.108					0.042					0.035					0.095				
Adj *R*^*2*^	0.133					0.139					0.104					0.039					0.031					0.092				
*F*(5,526*)*	41.6					43.7					32					11.7					9.46					27.758				

*Note*. N = 532. Only three decimals were reported for each value in the table. Significant results were reported in bold.

Cognitive Reappraisal was a significant positive predictor of all emotions of DPES. Suppression emerged as a significant negative predictor of all emotions of DPES, except for Amusement and Awe, which were not significantly predicted by Suppression.

## Discussion

This is the first study in which the relationship between specific discrete positive emotional tendencies and two of the major emotion regulation strategies of reappraisal and suppression was tested. To this end, we administered one stable measure of emotion regulation (ERQ)–already validated in Italian–and of discrete positive emotion dispositions (DPES), which was translated into Italian. Although DPES was intended to capture potentially universal, functionally distinct emotion constructs, the definitions of each emotion reflect English-language constructs. Therefore, we also tested the DPES factor structure, as well as its concurrent and divergent validity, in Italian. The Italian version of the DPES was validated through an analytic strategy that involved exploratory factor analysis and concurrent validity analysis. These steps yielded a six-factor scale, comprising 37 items out of the original 38 items, with acceptable to excellent alpha coefficients.

In this regard, some adjustments had to be made to reflect Italian culture. Specifically, the dimension of the original scales of Joy (items: 1, 2, 3, 4, 5, 6) and Contentment (items: 7, 8, 9, 10, 11) underlined the same dimension in this sample, which we labeled “Happiness.” Moreover, one item of the original Contentment scale (i.e., My life is very fulfilling) loaded on the Pride factor. These findings could be due to the “other-oriented” and interdependent nature of Italian culture as opposed to English-speaking societies [66]. While the first items of joy referred explicitly to joy, the other was related to satisfaction towards life and self-domain in general. Indeed, the concept of Joy in Italy should be based on interpersonal-relational elicitors more than on one’s own life [67]. However, no DPES items explicitly referred to as *interpersonal* situations. Therefore, it could be possible that participants did not recognize peculiar elicitors or prototypical situations of joy while reading the joy-related items.

Consequently, a macro factor encompassing all items of life fulfillment and life satisfaction, in general, emerged from a blend of Joy and Contentment. This scale was labeled “Happiness” since it entailed all aspects related to satisfaction with one’s own. Following the Contentment factor, Compassion and Love also showed a high frequency of occurrence, in line with the findings of previous studies on emotional lexicon usage in Italian samples [e.g., 68]. Finally, in this study, one Awe item (i.e., the mental shift: “I often look for patterns in the objects around me”) was removed due to its initial low communality with other items (.141), improving the fit the scale to our data. Finally, Pride had a high mean occurrence frequency. This result could be due to the renowned Italian disposition towards others [67], which could facilitate social comparison at the base of Pride. Finally, one item from the Pride original scale loaded on the general Happiness factor. It could be due to the semantic domain covered by this item “My life is very fulfilling,” which, in Italian, is close to the domain of self-accomplishment. The concurrent validity of the Italian-DPES scale was evaluated with PA and NA and the BFI traits. First, the results showed that Italian-DPES had moderate to strong positive correlations with positive affect and moderate correlations with negative affect. Specifically, all discrete emotions showed significant correlations with PA; only Amusement and Compassion showed positive (but not significant) correlation with Negative Affect.

Furthermore, all dispositions showed significant correlations with BFI personality traits. All factors were significantly positively correlated with Extraversion, in line with Shiota et al. [20]. On the other hand, while in Shiota et al. [20]. All DPES subscales correlated significantly also with Agreeableness, except for Amusement. All DPES scales correlated negatively with Neuroticism, except for Compassion, which is also in line with Shiota et al. [20]. Finally, all emotions displayed significant positive correlations with Openness to experience personality factors. Agreeableness predicted positively Happiness, Love, Compassion, and Awe, consistent with the claim of McCrae and Costa [69], who suggested that this trait facilitates positive affect. Conversely, highly conscientious people were less inclined to experience life situations as amusing and to feel the love in their daily activities. Conscientiousness is defined as the propensity to respect social conventions to control impulses and to achieve personal goals [70], and some evidence demonstrated that only certain specific amusing situations were positively associated with this personality trait (i.e., self-enhancing humor as a coping strategy). It would be relevant to investigate amusement-related elicitors that the participants considered while completing the scale. At the same time, Conscientiousness predicted Love negatively. This result is surprising since previous research demonstrated the positive role of Consciousness in determining different facets of love (i.e., intimacy, passion, commitment, relationship satisfaction) [e.g., 71]. Additionally, Shiota et al. [20] showed that Conscientiousness and Love are positively, even though not significantly, related. However, all these studies were conducted in English-speaking countries. A possible explanation of this finding is that the Italian culture defines love differently from other Anglo-Saxon cultures.

On the other hand, Conscientiousness predicted Compassion, Pride, and Happiness positively. Neuroticism predicted all emotions negatively except from Compassion, which is also in line with Shiota et al. [20]. Almost all emotions displayed significant positive correlations (and were predicted by) with Openness to experience personality factors, consistent with the literature on positive emotions. However, Openness to experience did not predict Happiness, and it predicted Love negatively. This was surprising since DPES measure a Love directed towards other people. Italian people are perhaps more inclined to address this Love only to their intimate partners and not to others.

Finally, and importantly, our results revealed significant correlational patterns between each emotion, suppression, and reappraisal emotion regulation strategies, in line with previous studies [14, 17, 18, 72]. Reappraisal promotes better management of stressful consequences; thus, it can increase the likelihood of experiencing positive states. Cognitive reappraisal was positively associated with all positive emotion tendencies, thus supporting its main adaptive evolutionary function. Crucially, cognitive reappraisal strongly predicted Happiness, Awe, and Pride. This result is in line with the existing literature on Joy and positive emotions-emotion regulation strategies in general [9, 24, 73] and suggests that Italian people may prefer reappraising situations in terms of happiness, pride, and awe, which usually trigger a cognitive restructuring people’ actual worldview. Indeed, reappraisal is an effective strategy also used to decrease positive affect [74]. Therefore, participants could have activated this process also to down-regulate positive emotions, that is, to diminish their intensity. For instance, the high uncertainty related to awe-inspiring situations [75] could have prompted participants to diminish its intensity and turn it into something more manageable and acceptable.

On the other hand, suppression also intervenes in modulating the outcome of emotional response at the expressive level; thus, we assumed, again, a causal link from this strategy and each emotion. In line with the literature on positive affect [13, 15, 18, 73, 76], we also found that suppression predicted Happiness, Pride, Love, and Compassion negatively and significantly. However, amusement was positively, but not significantly, associated with suppression. Maybe because Amusement is associated with both adaptive and maladaptive humorous responses to stressful situations [77–79]; thus, sometimes, Italian people may need to regulate it to fit the delicate demands of a social situation.

## Conclusions

In this study, for the first time, the relationship between specific emotion dispositions and the two emotion regulation strategies of reappraisal and suppression was tested. Each positive emotion tendency correlated positively with reappraisal and negatively with suppression, except for Amusement, which showed a positive and even non-significant correlation. This supported the adaptive value of cognitive reappraisal in the emotion regulation process. We also conducted a cross-cultural validation of the Dispositional Positive Emotions Scale in an Italian sample. The results showed an excellent internal consistency of this scale as well as a convergent validity with the PANAS factors. All BFI factors were significant predictors of almost all discrete emotions. Although these findings are promising, future research should consider also other cultural and contextual-specific factors to deepen these aspects. For instance, it could be useful to focus on different religious Italian samples, since some emotions, such as awe or compassion, can be related to this aspect [80, 81]. Specifically, it would be interesting to elucidate the peculiar situational elicitors of cognitive reappraisal of positive emotions, to understand when people tend to reappraise a situation as happy and thus capture cultural variations of this positive emotions. Specifically, DPES cannot connect peculiar positive emotion elicitors with systematic ER strategies. This can be a useful aspect to be deepened by future studies, for instance, by integrating also the Ecological Momentary Assessment or daily diaries.

Moreover, the final Italian DPES structure did not match the original English factor structure. Future studies should corroborate our finding and it could be useful to add other items to DPES to differentiate between Joy and Contentment in the Italian population. Further, our sample composed of women for the most part. Therefore, future validation studies should investigate the role of gender in emotion disposition and regulation. Finally, adopting a finer granularity of emotion regulation strategies (e.g., different reappraisals strategies [82]) to manage positive emotions could provide useful information, especially about the understudied Italian population. In this regard, the main discrepancies with previous studies could be explained in terms of cultural variations. This observation may support the assumption that emotions can be conceived as containing prototypical elements (*core themes*), even though their peripheral aspects can vary according to the context [4, 83]. Therefore, the present cross-cultural validation study may allow capturing both core emotional themes, and their variations also related to other phenomena, in this case, personality traits. This result maybe relevant also in the clinical field, since it could help clinicians design tailored interventions for people suffering from personality or affective disorders, in relation to the emotion regulation strategies they usually adopt to manage positive emotions [e.g., 84, 85]. In brief, the Italian DPES is easy to administer and can provide quick information on frequently experienced emotions and their link with other emotional or cognitive processes across a wide array of domains.

Overall, the findings demonstrated specific and strong links between cognitive and emotional depositions and evidenced that Italian DPES has good validity and reliability [86, 87] and is suitable to be used with an Italian sample.
